# Foodborne-associated *Shigella sonnei*, India, 2009 and 2010

**DOI:** 10.3201/eid1711.110403

**Published:** 2011-11

**Authors:** Suman Nandy, Shanta Dutta, Santanu Ghosh, Arindam Ganai, Jyothi Rajahamsan, Ramani Bai Josef Theodore, Nasira K. Sheikh

**Affiliations:** National Institute of Cholera and Enteric Diseases, Kolkata, India (S. Nandy, S. Dutta, S. Ghosh, A. Ganai); Medical College, Thiruvananthapuram, India (J. Rajahamsan, R.B.J. Theodore); Rajashree Chhatrapati Shahu Maharaj Government Medical College, Kolhapur, India (N.K. Sheikh)

**Keywords:** *Shigella sonnei*, foodborne outbreaks, antimicrobial resistance, bacteria, India, letter

**To the Editor:** Infection with *Shigella* spp. is a major cause of foodborne diseases, which have increased considerably during the past decades, but only a small fraction of cases are reported (*1*). *S. dysenteriae* and *S. flexneri* are the predominant species in the tropics; clinically, *S. dysenteriae* serotype 1 is associated with severe disease, large outbreaks, or epidemics. *S. sonnei* occurs more frequently in industrialized than in developing countries and causes milder illness than *S. dysenteriae* and *S. flexneri*. However, occasional foodborne outbreaks by antimicrobial drug–resistant *S. sonnei* have been reported from the United States, Japan, and European countries, mostly among children (*2**–**5*). During recent years, in Thailand, Vietnam, and Sri Lanka, the predominant species has shifted from *S. flexneri* to *S. sonnei*, a phenomenon possibly linked with country’s level of development (*6**,**7*). As a result, *S. sonnei* outbreaks are also being reported from developing countries (*8*). In India, the scenario differed somewhat. Devastating outbreaks of dysentery by multidrug-resistant *S. dysenteriae* type 1, with high case-fatality rates, affected major parts of the country during 1984–1985 (*9*). After a gap of 18 years, during 2002–2003, *S. dysenteriae* type 1 with an altered antimicrobial drug resistance pattern (100% fluoroquinolone resistance) reemerged, causing several dysentery outbreaks in West Bengal (*10*). Although *S. flexneri* was the major species, since 2005, *S. dysenteriae* type 1 has not been isolated (*9*).

We report 2 foodborne outbreaks of *S. sonnei* in India, 1 each from Kerala (southern part) in February 2009 and Maharashtra (western part) in February 2010, which support extension of *S. sonnei* into India. The outbreak isolates were characterized by antimicrobial drug resistance and plasmid and pulsed-field gel electrophoresis profiles.

On February 1, 2009, >300 persons (age range 2–70 years) attended a marriage party at Thiruvananthapuram, Kerala, where they were served local food made of rice, lentils, milk, and water. Within 12 hours after eating, ≈60% of persons had onset of acute diarrhea, vomiting, and abdominal pain and were admitted to local village or district hospitals or the nearest government general hospital for treatment. Illness was more severe in children; because of clinical severity, 10 children (<10 years of age) were admitted to a referral hospital for children in Thiruvananthapuram. One child (7 years of age) was moved to the pediatric intensive care unit because of altered sensorium and drowsiness and was treated with intravenous ceftriaxone and metronidazole. Others were treated with oral cefotaxime until recovery and were discharged. Fecal samples from 15 patients were processed at the local public health laboratory for enteric pathogens; 9 (60%) of 15 samples yielded *S. sonnei* as the sole pathogen. On microscopic examination, 12 (80%) samples had erythrocytes.

The second outbreak occurred on February 11, 2010, at Kolhapur, Maharashtra, among day laborers and their family members who had eaten in 1 madrasa (religious place). Approximately 150 persons reported diarrhea, vomiting, abdominal pain, and fever. They were admitted to the Government Medical College, Kolhapur, and treated with intravenous fluid (lactated Ringer’s solution), oral rehydration solution, intravenous ceftriaxone, and metronidazole. All patients were discharged after complete recovery. *S. sonnei* was isolated as the sole pathogen from 14 (70%) of 20 fecal samples or rectal swab samples from those patients.

Six isolates from outbreak 1 and 11 isolates from outbreak 2 were sent to the National Institute of Cholera and Enteric Diseases (Kolkata, India) for confirmation. We characterized those isolates to determine whether the outbreak isolates of *S. sonnei* predominant in India were clonal in origin.

Antimicrobial drug resistance profiles differed in the 2 outbreaks (Figure) when drug susceptibility was tested by disk diffusion. MICs of antimicrobial drugs by Etest (AB Biodisk, Solna, Sweden) were >32 µg/mL for tetracycline and co-trimoxazole, >256 µg/mL for nalidixic acid, and ≈4 µg/mL for norfloxacin and ciprofloxacin. Plasmid profiles of the isolates showed absence of large plasmids (212 kb) and several smaller plasmids arranged in distinct patterns in each group (data not shown). Because the isolates caused invasive diarrhea (erythrocytes in feces), large plasmids might have been lost through repeated subculture. DNA fingerprinting was performed by pulsed-field gel electrophoresis in a CHEF-DRIII system (Bio-Rad Laboratories, Hercules, CA, USA) after DNA digestion by *Xba*I following standard PulseNet protocol and by using *Salmonella enterica* serovar Braenderup H9812 as control strain. A few sporadic *S. sonnei* isolates from patients of the Infectious Disease Hospital, Kolkata, were included for comparison. The patterns were analyzed by using Dice coefficient, and a dendrogram was generated by using FP Quest Software (Bio-Rad). The isolates with ≈90% similarity threshold were grouped under 1 cluster. Distinct clusters were observed for outbreak 1 (cluster A), outbreak 2 (cluster B), and sporadic 2009 (cluster C) isolates, and patterns in each cluster differed by only a few (*1**,**2*) smaller bands.

**Figure:**
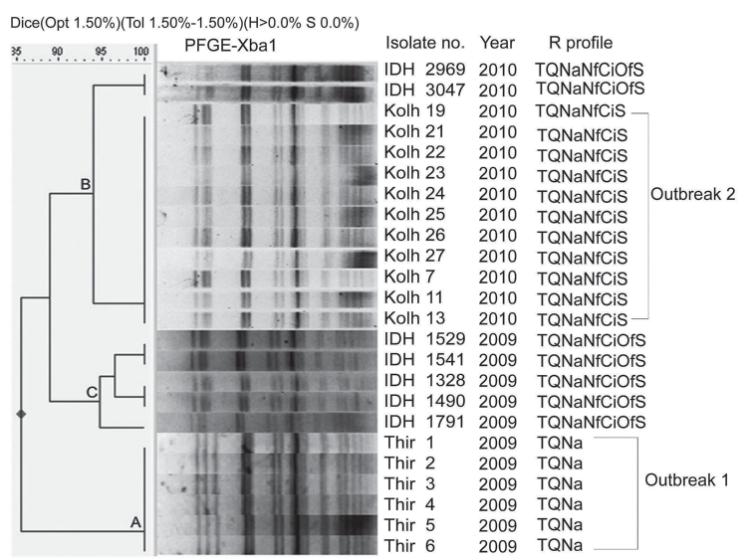
Digested pulsed-field gel electrophoresis (PFGE) profiles of *Shigella sonnei* outbreak isolates, India (Thiruvananthapuram, Kerala; Kolhapur, Maharashtra), by cluster analysis and comparison with sporadic isolates (IDH). Thir, isolates from Thiruvananthapuram, Kerala; Kolh, isolates from Ispurli, Shiroli Taluk, Kolhapur district, Maharashtra; IDH, isolates from Kolkata, West Bengal; R, resistance; T, tetracycline (30 µg); Q, co-trimoxazole (25 µg); Na, nalidixic acid (30 µg); Nf, norfloxacin (10 µg); Ci, ciprofloxacin (5 µg); Of, ofloxacin (5 µg); S, streptomycin (10 µg).

Therefore, our study supports emergence of *S. sonnei* outbreak clones in India during 2009–2010. These outbreaks may be the forerunners of many more *S. sonnei* outbreaks.
